# The Osteogenic and Tenogenic Differentiation Potential of C3H10T1/2 (Mesenchymal Stem Cell Model) Cultured on PCL/PLA Electrospun Scaffolds in the Absence of Specific Differentiation Medium

**DOI:** 10.3390/ma10121387

**Published:** 2017-12-04

**Authors:** Timothée Baudequin, Ludovic Gaut, Marc Mueller, Angela Huepkes, Birgit Glasmacher, Delphine Duprez, Fahmi Bedoui, Cécile Legallais

**Affiliations:** 1 CNRS, UMR 7338 Biomechanics and Bioengineering, Sorbonne Universités, Université de Technologie de Compiègne, 60200 Compiègne, France; timothee.baudequin@mail.mcgill.ca (T.B.); angela_huepkes@web.de (A.H.); 2 CNRS UMR 7622 IBPS-Developmental Biology Laboratory, Sorbonne Universités, UPMC Univ Paris 06, F-75005 Paris, France; ludovic.gaut@upmc.fr (L.G.); delphine.duprez@upmc.fr (D.D.); 3 Inserm U1156, F-75005 Paris, France; 4 Institute for Multiphase Processes, Leibniz Universität Hanover, D-30167 Hanover, Germany; mueller@imp.uni-hannover.de (M.M.); glasmacher@imp.uni-hannover.de (B.G.); 5 CNRS, UMR 7337 Roberval Laboratory for Mechanics, Sorbonne Universités, Université de Technologie de Compiègne, 60200 Compiègne, France; fahmi.bedoui@utc.fr

**Keywords:** scaffold, polymer, electrospinning, mesenchymal stem cell, cell differentiation, tissue engineering

## Abstract

The differentiation potential of mesenchymal stem cells (MSC) has been extensively tested on electrospun scaffolds. However, this potential is often assessed with lineage-specific medium, making it difficult to interpret the real contribution of the properties of the scaffold in the cell response. In this study, we analyzed the ability of different polycaprolactone/polylactic acid PCL/PLA electrospun scaffolds (pure or blended compositions, random or aligned fibers, various fiber diameters) to drive MSC towards bone or tendon lineages in the absence of specific differentiation medium. C3H10T1/2 cells (a mesenchymal stem cell model) were cultured on scaffolds for 96 h without differentiation factors. We performed a cross-analysis of the cell–scaffold interactions (spreading, organization, and specific gene expression) with mechanical (elasticity), morphological (porosity, fibers diameter and orientation) and surface (wettability) characterizations of the electrospun fibers. We concluded that (1) osteogenic differentiation can be initiated on pure PCL-based electrospun scaffolds without specific culture conditions; (2) fiber alignment modified cell organization in the short term and (3) PLA added to PCL with an increased fiber diameter encouraged the stem cells towards the tendon lineage without additional tenogenic factors. In summary, the differentiation potential of stem cells on adapted electrospun fibers could be achieved in factor-free medium, making possible future applications in clinically relevant situations.

## 1. Introduction

Electrospun fibers are promising scaffolds for tissue engineering and regenerative medicine with a growing interest over the last decade [1,2,3,4]. Compared to other fiber production processes (such as phase separation, extrusion or self-assembly [5]), electrospinning has several advantages for both in vitro and in vivo studies. The electrospinning method is relevant at the laboratory scale for prospective studies (making possible custom-made devices) and for further mass production [5,6]. This method is highly versatile and tunable [4]. Depending on the choice of materials and procedures, electrospun scaffolds offer various degradation rates [5], as well as pore interconnectivity [6], high porosity [6], a high surface/volume ratio [4,5,6] and anisotropy at the molecular level in the case of fiber alignment [1,7,8]. These properties are well-known as ways of controlling cell adhesion and cell behavior [6,8,9]. Moreover, the submicrometric diameter of the electrospun fibers is similar to that of collagen fibers in natural extracellular matrix [1,10], establishing a biomimetic environment for cells. Electrospun scaffolds also show appropriate and adjustable mechanical properties, compared to other laboratory-made synthetic matrices [1,10], due to a stable structure with a smaller number of defects [5,6].

Many different electrospun polymer solutions have been studied as biomaterials to promote organ/tissue regeneration or repair, such as components of the musculoskeletal system, bone, tendon, ligament [11,12,13,14,15] or dental applications [16]. Polylactic acid (PLA), polycaprolactone (PCL) and polyglycolic acid (PGA) are frequently cited as the most commonly-used polymers and are approved by the Food and Drug Administration (FDA) [4,17]. PCL scaffolds have been extensively used for bone tissue engineering, in combination with the use of growth and differentiation factors or composite solutions [4,14,18]. In contrast, pure PLA has been shown to be the most relevant polymer for the development of tendon substitutes in comparison with PCL [19], PGA [20] or poly(lactic-co-glycolic) acid (PLGA) [20]. Moreover, the tunable morphological and mechanical properties of electrospun scaffolds are of primary interest as tendon regeneration is particularly dependent on these signals [11,13,21].

Mesenchymal stem cells (MSC) are multipotent stem cells that are able to differentiate into various lineages including muscle, bone, cartilage and tendons/ligaments with a specific differentiation medium [22,23,24]. The differentiation potential of MSCs has been extensively tested on different scaffolds. However, this potential is often assessed with lineage-specific differentiation media [25], making it difficult to interpret the real contribution of the properties of the scaffold in the differentiation process. Moreover, it can be important to remove additional chemical factors from the culture processes to avoid undesired effects in vivo after reaching the clinical stage [26,27]. The choice of biomaterial is effectively crucial for tendon, ligament or enthesis (tendon/ligament to bone interface) reconstruction [28,29]. We hypothesize that analyzing the properties of the biomaterials that drive cell lineage differentiation potential should be devoid on culture medium parameters. In this study, we analyzed the ability of different PCL/PLA electrospun scaffolds to drive MSCs towards bone or tendon lineages in the absence of specific differentiation media. C3H10T1/2 cells [30] are able to differentiate towards different cell lineages in controlled environments, including bone and tendon, and are frequently used as a model for studies at their early stage of development [25,31,32,33]. Cultures conducted on scaffolds were compared to cells maintained in regular culture conditions (flat culture substrate, proliferation medium without factors) to highlight specifically the intrinsic effects of the electrospun fibers’ properties on the biological response.

## 2. Results

**Figure 1:**
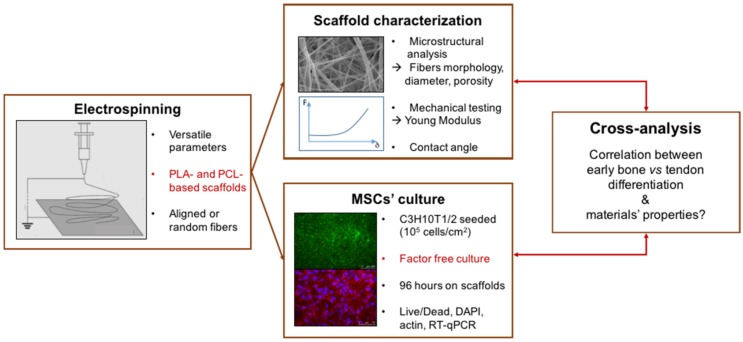
Design of the present study. The osteogenic and tenogenic potential of PCL- (polycaprolactone) and PLA-based (polylactic acid) electrospun scaffolds was investigated in the absence of specific differentiation media with a cross-analysis of biological, morphological and mechanical behaviors.

In order to test the ability of different biomaterials to drive MSC differentiation towards a specific lineage, we manufactured electrospun fibers based on PCL and PLA solutions with different compositions (pure or blended solutions) and structures (random or aligned fibers) and analyzed the bone/tendon differentiation of MSCs cultured on these biomaterials. The settings for different scaffolds are summarized in Table 1. Polymer solutions and fiber structures were chosen to analyze the respective effects of the production parameters and their combinations. To allow comparison and avoid variability, we applied the same mechanical characterization to all the scaffolds, in addition to using similar cell culture protocols (Figure 1).

### 2.1. Electrospun Fiber Morphology

**Figure 2:**
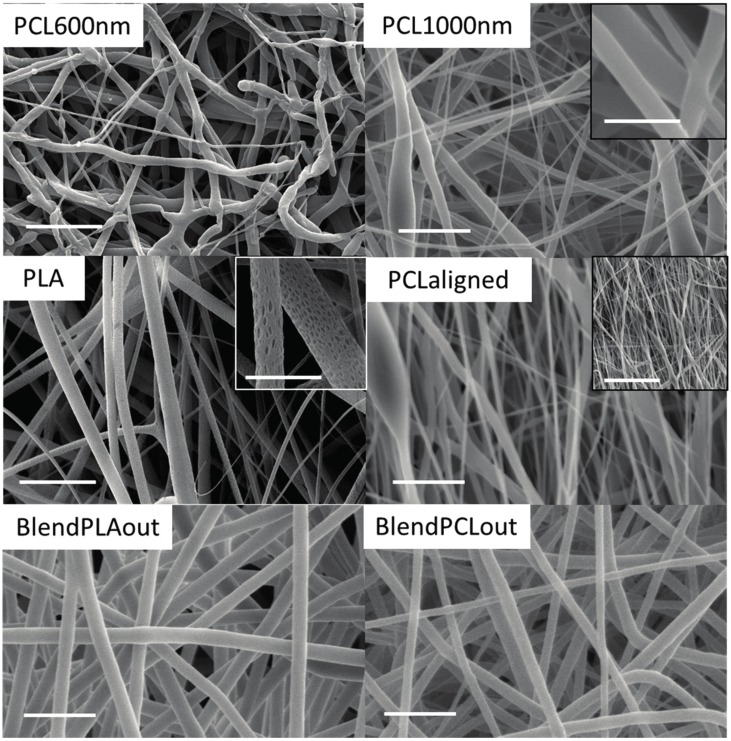
Scanning Electronic Microscopy (SEM) observations of the electrospun scaffolds. Insets in PLA, PCL1000nm and PCL-aligned panels: SEM observation of the PLA and PCL1000nm scaffolds to highlight porosity at the fiber scale, and of the PCL-aligned scaffold to highlight alignment. Main scale bars: 10 µm, PLA inset: 2 µm, PCL1000nm inset: 4 µm, PCL-aligned inset: 50 µm.

Scanning electronic microscopy (SEM) observations highlighted that all the electrospun fibers were generally homogenous in size and devoid of bead formation for all the polymers used (Figure 2). The fibers were deposited randomly with a rotating speed of 50 rpm, while they were aligned when the speed reached 1000 rpm (PCL-aligned scaffold). The change in angle between the fibers and the direction of rotation was measured by using Axio Vision software (Carl Zeiss, Oberkochen, Germany), the lower the angle the higher the fiber alignment). Unaligned fibers showed a mean angle of 15.5° ± 3.1° whereas aligned fibers showed a mean angle of 1.8° ± 1.8°. PLA scaffolds presented an upper layer of fibers less dense than the other scaffolds, which led to a macroscopic fluffy aspect with beads in some locations. This aspect didn’t induce additional degradation in aqueous environment during culture. Moreover, the surface of the PLA fibers was itself rough, or even nanoporous, as seen with a higher SEM magnitude (inset on Figure 2). This specificity has already been observed in the literature and may be explained by the high volatility of dichloromethane [34,35]. However, no effect on the cell behavior has been detected, possibly because the pore size is below the cellular detection threshold [36].

**Figure 3:**
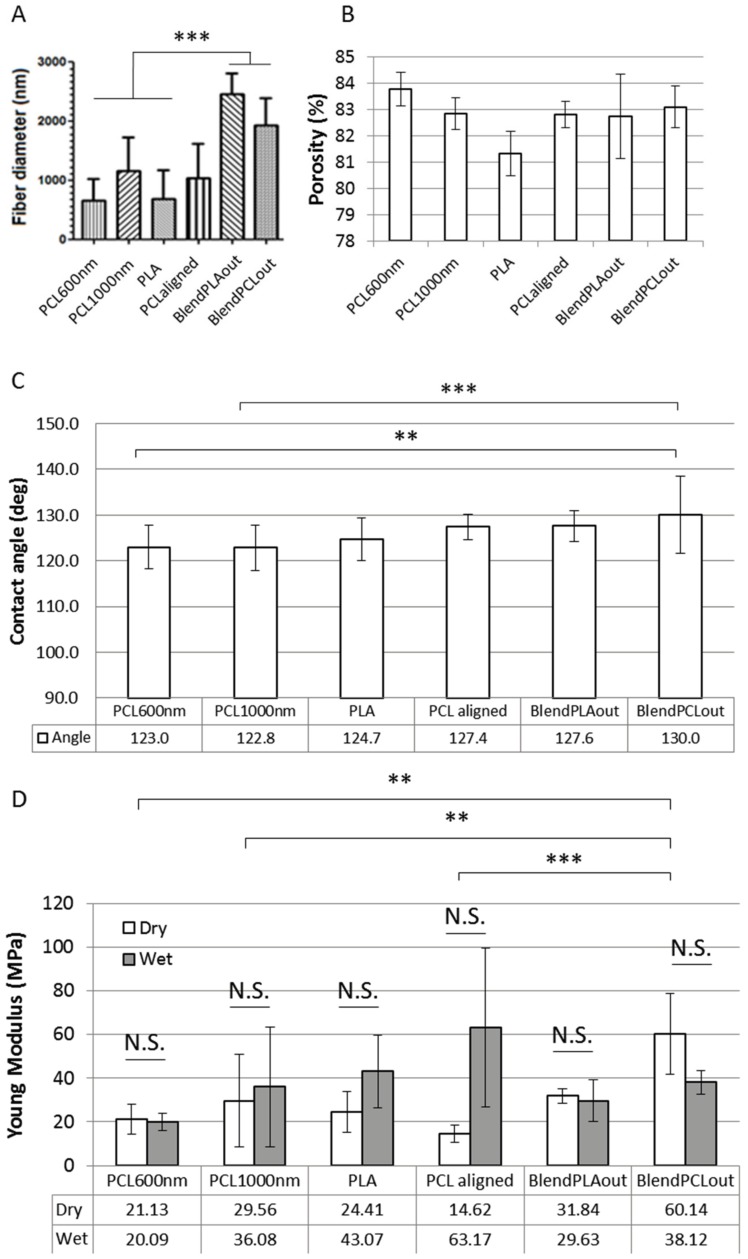
Morphological and mechanical characterization of the different scaffolds. (**A**) Fiber diameters of the electrospun scaffolds; (**B**) Estimation of scaffold porosity using image processing for the electrospun scaffolds. The µ + δ threshold focuses on the porosity of the upper layers of fibers; (**C**) contact angle measured on each electrospun scaffold; (**D**) Young’s moduli of each scaffold. Comparisons of Young’s moduli were made between dry and wet samples, and between scaffolds based on dry samples. N.S., non-significant, ** *p* < 0.01, *** *p* < 0.001.

The fiber diameters of each scaffold were measured on SEM images. Based on the diameters of the fibers, the scaffolds were sorted into two groups. The fiber diameters of pure PCL and pure PLA scaffolds ranged from around 500 to 1000 nm (Figure 3A). Coaxial blends had diameters of 2000 nm or more, significantly different from the other scaffolds (*p* < 0.001), with a maximum of 2461 ± 353 nm for BlendPLAout (Figure 3A). Globally speaking, the diameters were in the same range as those obtained classically using the electrospinning technique [37,38,39].

### 2.2. Surface Characterization

The porosity assessments at the scaffold scale (pores induced by the space between the fibers) are reported in Figure 3B. The original SEM images were converted to black and white for various binarization thresholds based on the mean (µ) and standard deviation (δ) of the image histogram [40,41]. The porosity was estimated from the black pixel (void)/white pixel (material) ratio. Three thresholds were studied (µ + δ; µ; µ − δ), but the results presented here focus on the µ + δ threshold, which only took into consideration the upper fiber layers after binarization. This threshold appears to be the most relevant for analyzing cell response because the cells are mostly in contact with the external fibers layers. These values can therefore be used to compare the morphology of the different scaffolds although only an estimation of the porosity of the whole scaffold could be derived [40,41,42,43,44,45]. The porosity varied from 80% to 84% among the different scaffolds (Figure 3B). The statistics information is not shown in the figure for readability reasons. Except for blended scaffolds, the differences between the scaffolds were found to be statistically significant (*p* < 0.05 to *p* < 0.001). Contact angle measurements were carried out on the surface of the dry electrospun fiber network. The results are summarized in Figure 3C. The scaffolds showed hydrophobic behavior with mean results from 122.7° (PLA) to 130.0° (BlendPCLout).

### 2.3. Mechanical Characterization of Electrospun Fibers

In order to characterize the mechanical properties of each scaffold, samples were submitted to tensile tests in dry or wet conditions. Wet conditions were defined as being after humidification in demineralized water. Young’s moduli of each scaffold are reported in Figure 3D. Humidification of the scaffolds prior to measurement did not induce significant changes in Young’s modulus values. Some samples showed a trend for a higher Young’s modulus in wet conditions. Although such an increase can occur in polymeric samples [46], this behavior was not found to be significant here. It can be seen as a lack of overall effect of the humidification with an increase in data dispersion. It is particularly noticeable for PCLaligned samples, were the aligned layers of fibers could have more easily detached from each other after humidification and therefore increased the variability of the measurements. In dry conditions, blended scaffolds showed the highest stiffness with a maximum of 60.14 ± 18.52 MPa for BlendPCLout. This value was statistically different from all the other pure PCL scaffolds (*p* < 0.001 to *p* < 0.01), however PCL600nm, PCL1000nm and PCLaligned were not statistically different from each other. As the Young’s Modulus has been shown to be linked to the fiber morphology [34,39,47], the differences in diameter from one PCL scaffold to the other could be here too slight to show differences in stiffness. There was no statistical difference between BlendPCLout and BlendPLAout (reverse coaxial structures). One can hypothesize that the overall stiffness of the blended fibers led to the same behavior regardless of the respective location of PCL and PLA in the core/shell studied structures. This mechanical characterization was meant to derive qualitative estimation of the stiffness of the scaffolds because fiber morphology, porosity and grip effects were not taken into consideration. Nevertheless, we estimate that this evaluation of mechanical properties was relevant for comparing scaffolds before cross-analyses of the biological response (Table 2).

### 2.4. Cell—Scaffold Interactions

**Figure 4:**
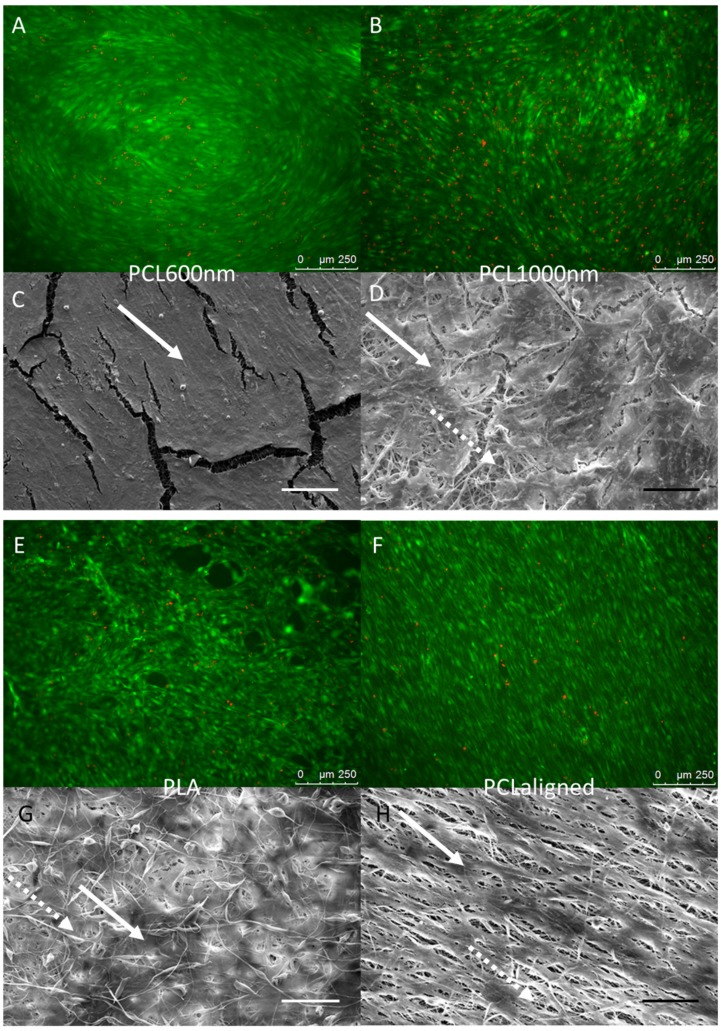

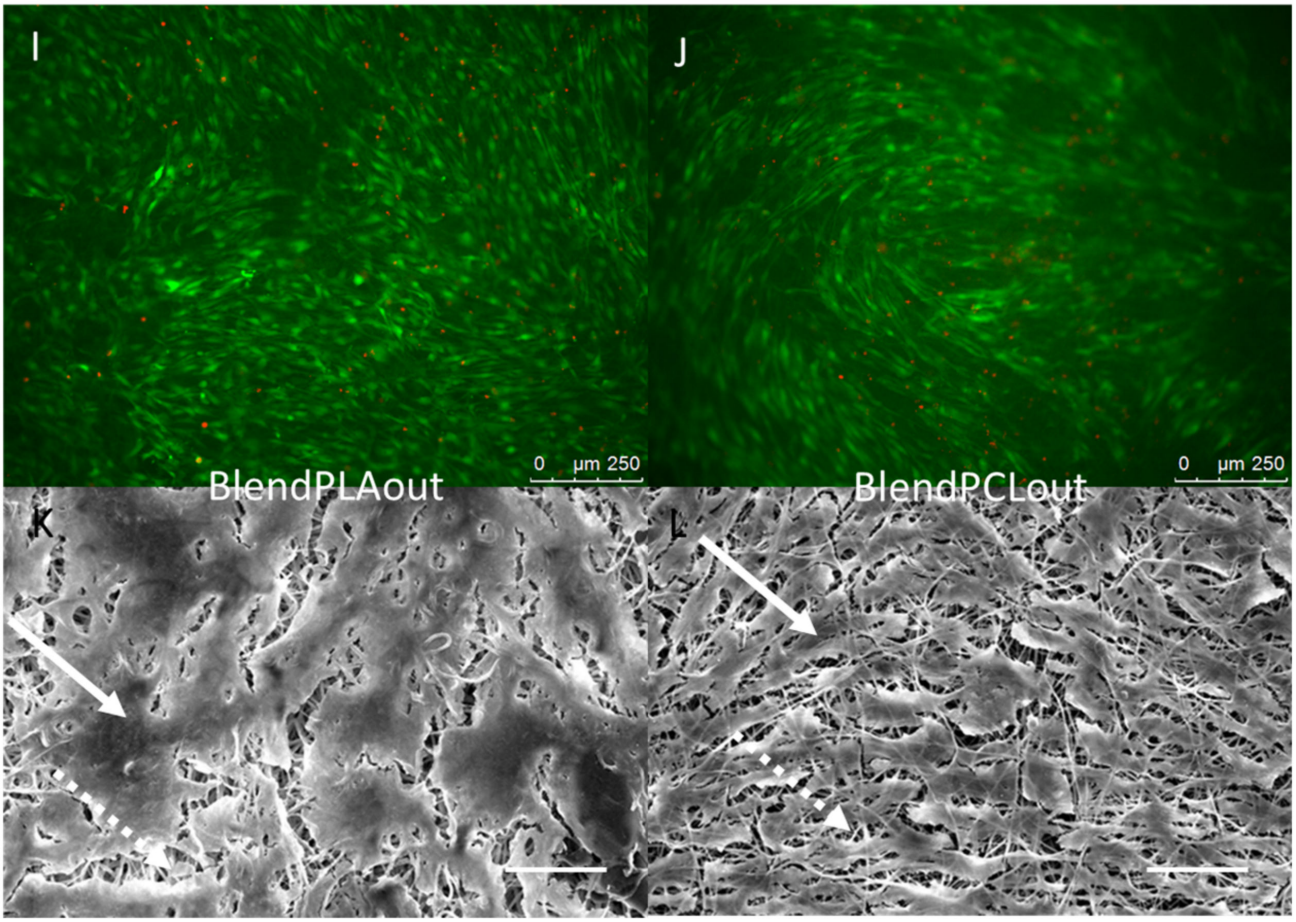
Fluorescence microscopy (**A**,**B**,**E**,**F**,**I**,**J**) and SEM observations (**C**,**D**,**G**,**H**,**K**,**L**) of C3H10T1/2 cells cultured for 96 h on PCL60nm (**A**,**C**), PCL1000nm (**B**,**D**), PLA (**E**,**G**), PCLaligned (**F**,**H**), BlendPLAout (**I**,**K**) and BlendPCLout (**J**,**L**) electrospun scaffolds. Fluorescence: Living cells (Calcein AM, green) and dead cell nuclei (EthD-1, red), scale bars 250 µm. SEM: scale bars 100 µm. Solid arrows: continuous cell tissue. Dashed arrows: fibers network visible due to cracks in the cell tissue or to a lower cell density.

**Figure 5:**
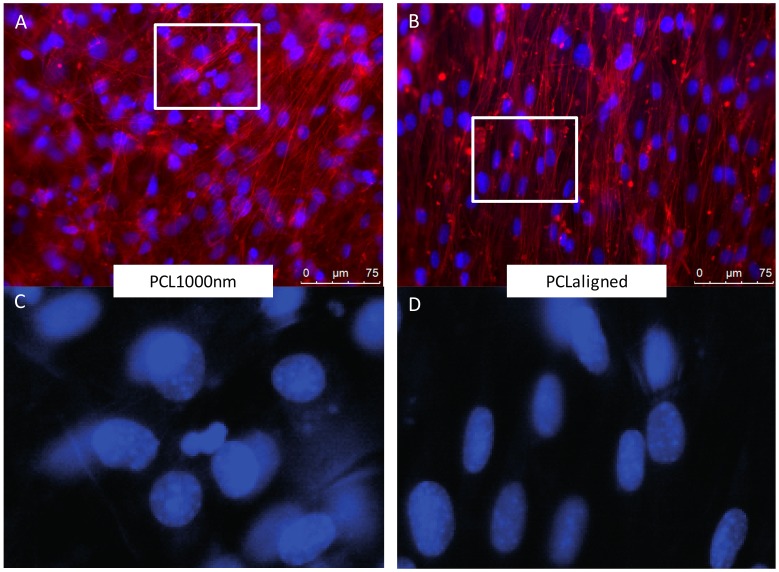
Fluorescence microscopy observations of C3H10T1/2 cells cultured for 96 h on PCL1000nm (**A**,**B**) and PCLaligned (**C**,**D**) scaffolds. (**A**,**C**) Actin filaments visualized with rhodamine-phalloidine (**red**) and nuclei visualized with DAPI (**blue**). (**B**,**D**) higher magnifications of nuclei (DAPI) of region of interest (white rectangles in **A**,**C**).

C3H10T1/2 stem cells were cultured for 96 h on the different electrospun scaffolds. C3H10T1/2 cells were plated at the same density on each scaffold and cultured in the same conditions with the same culture medium. A “Live/Dead” staining was used to observe living cells (Calcein AM, green) and dead cell nuclei (EthD-1, red) simultaneously (Figure 4A,B,E,F,I,J). The observations showed that all the scaffolds made possible the development of generally continuous and homogeneous tissue made from living cells covering the whole electrospun fiber network. The qualitative analysis of these images allowed us to conclude on excellent cell attachment and viability on all samples as a small number of dead cells was noticed. SEM observations further confirmed the development of a continuous cell layer. Cracks observed in several SEM images (Figure 4C,D,K,L) probably occurred during the preparation of the SEM samples as they were not seen in the fluorescence staining images of the cells. The fibers themselves appeared broken under the living tissue (Figure 4C). Cells cultured on pure PCL scaffolds showed the most continuous, dense and homogeneous tissue (Figure 4A,B,F). On the PLA scaffold, the floating upper electrospun fibers (Figure 2) led to the development of cells under these fibers, “inside” the scaffold (Figure 4G). Cell coverage thus appeared less dense on PLA-based than on pure PCL-based scaffolds, with some heterogeneous spots (Figure 4E). The observations for PCLaligned samples suggested an alignment of the cells (Figure 4F,H). Cell orientation was confirmed with the visualization of nuclei using DAPI simultaneously with actin filament staining (Figure 5A,B). The nuclei of C3H10T1/2 cells displayed an elliptic shape and were oriented parallel to the aligned actin filaments, in turn in the same orientation as the alignment of PCLaligned fibers (Figure 5B,D). In contrast, nuclei of cells cultured on PCL1000nm were round-shaped, consistent with an absence of any specific spatial organization (Figure 5A,C).

### 2.5. Cell Differentiation

**Figure 6:**
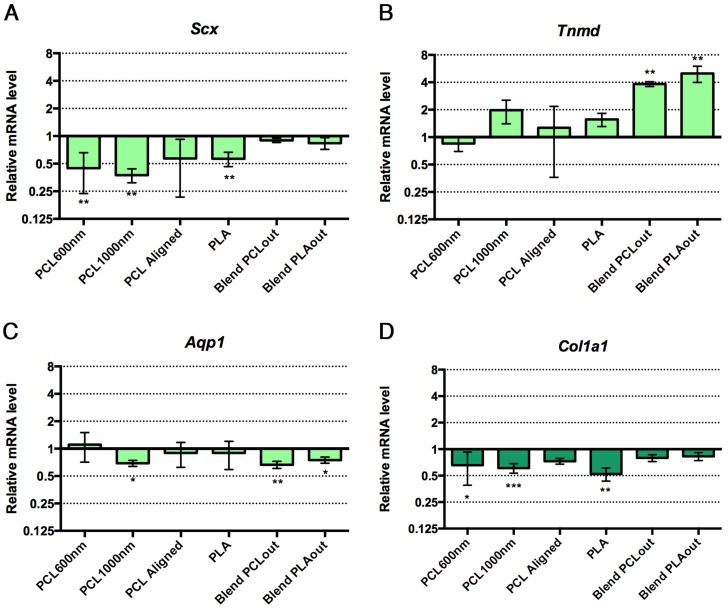

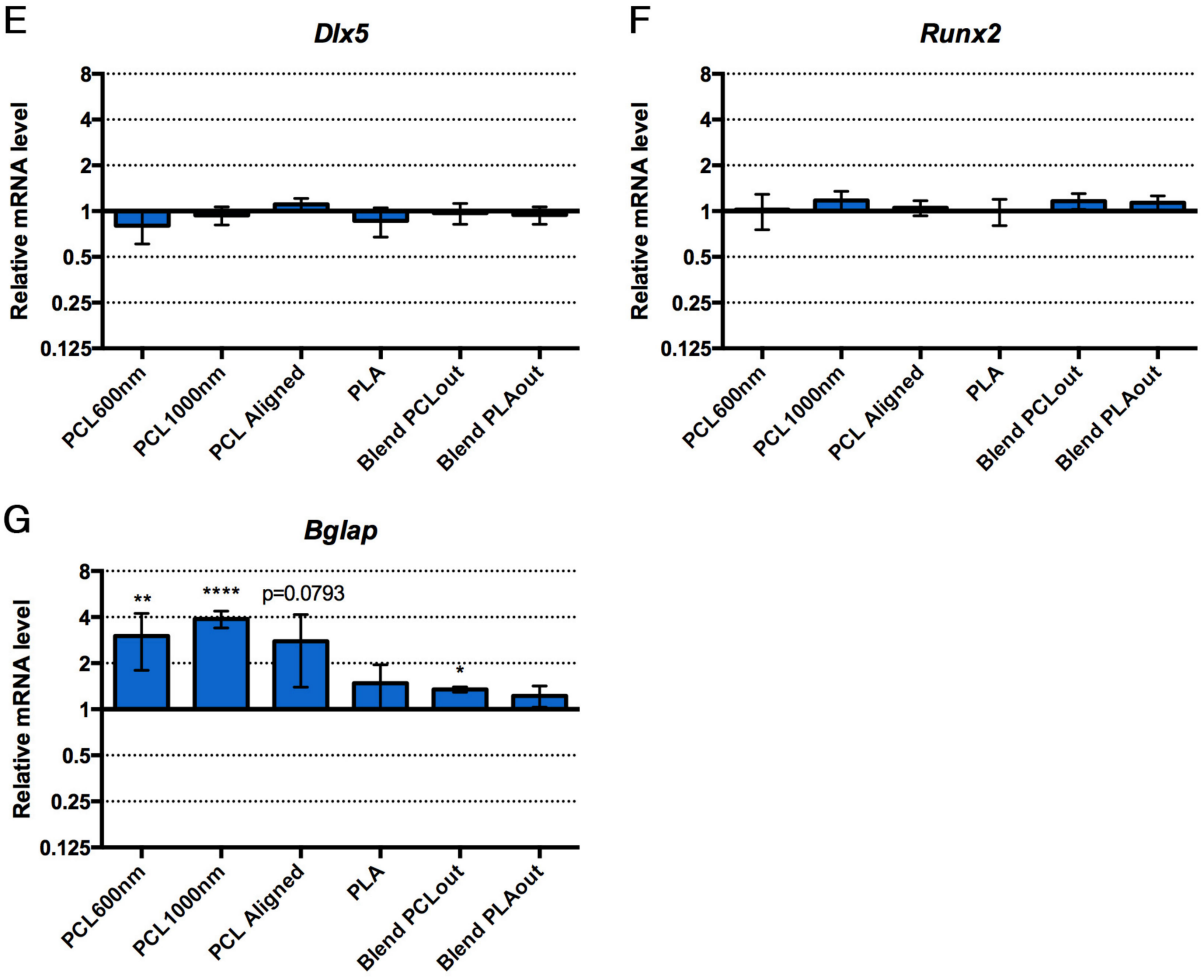
Relative expression of mRNA levels for tendon-related genes, Scx (**A**); *Tnmd* (**B**); *Aqp1* (**C**); (**green bars**) and bone-related genes, *Dlx5* (**E**); Runx2 (**F**); *Bglap* (**G**); (**blue bars**) in C3H10T1/3 cells cultured on the different electrospun scaffolds compared to control conditions (cells without scaffold). *Col1a1*, a component of tendon and bone and is represented in dark green bars (**D**). Control conditions have been normalized to 1. Error bars represent s.d. * *p* < 0.05, ** *p* < 0.01, *** *p* < 0.001, **** *p* < 0.0001.

The preferential differentiation outcomes of C3H10T1/2 cells cultured in the different electrospun scaffolds were evaluated with the expression of genes of interest by Reverse Transcription quantitative Polymerase Chain Reaction (RT-qPCR) at the end of 96 h of culture. Tendon- and bone-related markers were simultaneously analyzed in cells cultured in each scaffold. *Dlx5*, a transcription factor regulating the activation of specific bone markers [48], Runx2, a specific bone transcription factor [49], and *Bglap* (also called Osteocalcin) a late marker involved in bone mineralization [49] were used as bone-related genes. To assess to what extent stem cells were committed to the tendon lineage, we used Scx (Scleraxis), a bHLH (basic helix-loop-helix) transcription factor expressed in tendon progenitors and differentiated cells [50,51], *Tnmd* (Tenomodulin) a late tendon-specific marker [50,51] and *Aqp1* (Aquaporin-1) a marker for tendon development [32]. The expression of the gene *Col1a1* was also analyzed, although we are aware that *Col1a1* is expressed in both bone and tendon tissues. The relative mRNA levels of bone and tendon genes in stem cells cultured with the different scaffolds are presented in Figure 6. Cells cultured in regular conditions (flat glass substrate, proliferation medium without any factors) were used as a control group to express increases and decreases caused specifically by the presence of the scaffolds in the other groups. PCL scaffolds (PCL600nm, PCL1000nm (significant) and PCLaligned (non-significant)) led to an increase in *Bglap* expression (up to four-fold increase, significance *p* < 0.01 to *p* < 0.0001), associated with decreased expression of the Scx tendon-related gene (*p* < 0.01) compared to the control plastic cultures, suggesting a shift toward bone differentiation (Figure 6A,G). Both PCL/PLA blended scaffolds induced stem cells towards tendon differentiation, as assessed by a significant increase in *Tnmd* expression (up to four-fold increase, *p* < 0.01, Figure 6B). Based on the decrease in Scx and *Col1a1* expression (*p* < 0.01), and the absence of bone marker modifications, we conclude that PLA was not favorable for tendon or bone differentiation (Figure 6A,D).

## 3. Discussion

In the present study, mouse C3H10T1/2 mesenchymal stem cells [30] were cultured on various PCL and PLA electrospun scaffolds in the absence of specific differentiation medium. The use of identical culture protocols (no addition of osteogenic or tenogenic differentiation factors) for C3H10T1/2 cells seeded on different materials over a short period (96 h) as well as undifferentiated control groups without scaffolds highlighted which biomaterials were preponderant in bone or tendon differentiation from a multipotential stem cell population. In particular, mechanical properties and fiber morphology were evaluated to perform a cross-analysis with biological behavior. The identification of scaffolds allowing mesenchymal stem cells to differentiate into bone and tendon lineages could lead to the development of new constructs for the regeneration of enthesis [52,53].

All the various settings made possible the formation of the electrospun fibers (Figure 2) with similar hydrophilic profiles (Figure 3). Data summarizing both biological and mechanical characterizations of the six scaffolds used in this study are shown in Table 2. Pure PCL-based scaffolds favor cell differentiation towards bone differentiation based on the increase in *Bglap* expression, a late bone marker involved in mineralization [48], while coaxial blend materials favor tendon differentiation based on an increase in *Tnmd* (Figure 6). *Bglap* upregulation reached a 4-fold increase compared to non-specific culture conditions with a strong significance (*p* < 0.001) on PCL1000nm samples. This initiation towards bone differentiation of stem cells cultured with PCL scaffolds is consistent with the literature data where it is described that PCL is widely-used for bone regeneration [14,18]. However, in most cases, bone differentiation was observed in the presence of osteogenic medium including dexamethasone, ascorbic acid, or beta-glycerosphosphate [14,49]. Therefore, the differentiation effect may not be attributed to the material itself. In the present study, random scaffolds (PCL600nm, PCL1000nm) and PCLaligned (to a lesser extent) pushed stem cells towards bone differentiation, while decreasing tendon differentiation. This finding is of major interest for the design of bone tissue engineering protocols that do not involve additional growth and differentiation factors, in order to avoid clinical complications (such as ectopic bone formation) and regulatory issues [26,27].

Scaffolds with aligned structures have been reported as pushing stem cells towards the tendon cell lineage [54]. On the PCLaligned scaffold, the nuclei and cytoskeleton of stem cells were found to be aligned along the same orientation of the electrospun fibers (Figure 5B,D). However, this orientated cell morphology did not lead to significant modification in tendon gene expression (Figure 6A–C,E). It has nevertheless been shown that the alignment of polymeric structures promotes the ligament lineage after a long culture time (7 to 14 days [50,55]); this suggests that the alignment of PCL fibers was not an efficient way of modifying stem cell differentiation towards the tendon lineage in short term cultures.

In contrast with cell behavior on pure PCL samples, the expression of the tendon-related gene *Tnmd* was upregulated in the presence of both PLA and PCL polymers (BlendPCLout and BlendPLAout scaffolds). The 4-fold increase in *Tnmd* expression compared to control was related to the same 4-fold upregulation obtained with Egr1-C3H10T1/2 cells compared to C3H10T1/2 cells in a previous study [25]. This similar range of increase of *Tnmd* expression upon Egr1 over-expression allowed us to conclude on a significant effect of the blended scaffolds on the stem cell fate towards tenogenic differentiation. Moreover, *Bglap* expression (assessing bone differentiation) was not significantly increased in these mixed PLA/PLC scaffolds, regardless of whether PLA or PCL was electrospun as the external layers (Figure 6). SEM observations of fiber networks did not reveal any morphological difference between either coaxial blend scaffolds (Figure 2). Moreover, PCL and PLA are not expected to mix during the fabrication process as seen in previous validation studies [56]. As the stem cells did not homogeneously cover the pure PLA scaffold (Figure 4E,G) and did not show any sign of either tendon or bone differentiation (Figure 6), we hypothesized that the presence of PCL provided high adhesion and proliferation potential while PLA enhanced the expression of the tendon-related genes after the state of confluence was reached. We concluded therefore that PCL and PLA acted together to initiate stem cell differentiation towards the tendon lineage: combining both polymers made it possible to reach optimal surface coverage followed by differentiation towards the tendon lineage in the absence of any specific factors. Triggering differentiation at short term (96 h), especially late markers such as *Tnmd* or osteocalcin, was an interesting result to obtain rapidly functional tissue engineering constructs in a clinical perspective. We hypothesized that this was achieved here by the combination of favorable chemical (polymer nature), biological (rapid proliferation and dense population) and mechanical (porosity, topography and alignment) environmental signals. We avoid completely the use of additional differentiation factors in this study to be relevant with clinical requirements [26,27]. As a comparison, literature suggests that pre-osteoblastic cells in differentiation culture conditions (without scaffold but with bone-promoting factors, from ascorbic acid only to 3-factor cocktail or novel chemicals) usually needs a longer culture time (10–12 days or more) to reach maturation [57,58,59].

It has to be mentioned that the nature of the polymer is probably not the only parameter that alters stem cell fate. The effect of adding PLA to PCL cannot be distinguished from the influence of fiber morphology, as the coaxial scaffolds led to the formation of the biggest fiber diameters in our samples (Table 2, Figure 3) with good homogeneity. According to our results, a linear correlation (R2 = 0.95, MS Excel linear regression tool on *Tnmd* results) established a link between a high fiber diameter (around 2 µm) with the tenogenic differentiation potential of C3H10T1/2 cells. This trend is consistent with the findings of Cardwell et al. [60], who demonstrated that fiber diameter was a preponderant parameter for monitor stem cell differentiation towards tendon lineage. The non-monotonic response of bone cells to scaffold topography has already been noted, especially on PCL [36,61,62,63].

A strong proportionality relationship has been reported in the literature between fiber diameters and the Young moduli of scaffolds in the case of electrospun samples [34,39,47]. This proportionality can also be observed in our results but was not found to be significant, and a clear linear correlation didn’t appear. We hypothesized that the differences in structure (alignment with multi-layers, coaxial composites) altered the proportionality relationships. However, the discussion on the effect of fiber diameter reported earlier could thus be conducted in terms of scaffold elasticity with the same conclusions. The elasticity values of electrospun materials showed considerable variability in the literature [18,38,64,65] which could be explained by changes in the polymer solution but also in technical procedures. We found here a Young’s modulus from 15 MPa to 60 MPa for the dry materials, i.e., consistent with the medium-range values found in literature (Figure 3).

In summary, the nature of the polymer and the morphology of the electrospun scaffolds could act together to monitor the fate of stem cells grown on the surface without additional specific differentiation media. A bone lineage profile was observed on various pure PCL scaffolds, while the presence of both PCL and PLA, combined with a fiber diameter of more than 2000 nm, initiated tendon differentiation in C3H10T1/2 cells. Further investigations could focus on the eventual synergetic effects that may occur between the signals with the most influence. These results are of interest for the regeneration of the musculoskeletal system. This factor-free process could also help anticipate the behavior of a patient’s own cells that would colonize the biomaterial implanted alone. There are still few tissue-engineered substitutes combining cells and scaffolds that have reached clinical trials. It should be noted that all the results analyzed here reflect the primary response of the C3H10T1/2 mesenchymal stem cells. These cells are frequently used as a MSC model to reveal the potential of various processes on the stem cell fate, including on electrospun scaffolds, for early-development studies [25,32,33]. Differentiation to one or other lineage needs to be assessed over longer culture periods and gene expression confirmed by proteomic studies to ensure that a mature state is reached before implantation. Following the screening of various electrospun fibers configurations reported in the present article using a MSC-like cell model, confirmation and further applications on the most promising scaffolds for each lineage will have to be performed with primary stem cells.

## 4. Materials and Methods

### 4.1. Materials

The PCL granules (Mw 70–90 kDa) were obtained from Sigma-Aldrich, St. Louis, MO, USA. The PLA powder (Mw 59–101 kDa, low viscosity) and granules (Mw 150 kDa) were obtained from Sigma-Aldrich, St. Louis, MO, USA (for the pure PLA scaffolds) and from Natureplast, Ifs, France (for the blends), respectively. Chloroform (CHCl_3_, Sigma-Aldrich, St. Louis, MO, USA), dichloromethane (DCM, CH_2_Cl_2_, VWR International, West Chester, PA, USA), acetone (C_3_H_6_O, Labgros, France) and 2,2,2-trifluoroethanol (TFE, ABCR, Karlsruhe, Germany) were used as solvents.

### 4.2. Electrospinning Process

Two custom-made electrospinning devices were used at the Université de Technologie de Compiègne (UTC) and the Leibniz University of Hannover, with respectively a rotating drum collector (diameter 75 mm) from Nabond (Shenzhen, China) and a homemade drum (diameter 150 mm).

The technical parameter sets for each scaffold were tuned by adjusting polymer and solvent concentration, flow rate and voltage with a trial and error approach until optimal morphology of the fibers was obtained (i.e., homogeneous fiber diameter without beads). These final parameters, as well as the nomenclature, are summarized in Table 1.

Coaxial scaffolds were prepared with a homemade double-lumen needle. BlendPCLout scaffolds were prepared with pure PCL in the outer shell of the fiber and a blend of PCL/PLA in the core. In the case of BlendPLAout, the blend of PCL/PLA was in the outer shell and PCL in the core.

PCL was spun as aligned (PCL-aligned) or random fibers (PCL600nm, PCL1000nm). Random fibers were prepared with a rotating speed of 50–250 rpm and aligned fibers (PCL-aligned) with a rotating speed of 1000 rpm.

Pure PLA scaffolds were prepared at the UTC Compiègne; BlendPCLout, BlendPLAout, PCL-aligned and PCL1000nm scaffolds were prepared at the Institute for Multiphase Processes, Leibniz University of Hannover, Hanover, Germany. The PCL600nm scaffold samples were a gift from the University of Strasbourg, ICPEES, Strasbourg, France.

### 4.3. Morphological Characterization of the Scaffolds

Fiber morphology and mean diameter were evaluated with scanning electronic microscopy (SEM, Quanta FEG 250, FEI, Hillsboro, OR, USA). Samples were coated with gold before observations. Scaffold porosity was estimated using the method described by Ghasemi-Mobarakeh et al. [40]. Briefly, SEM images were converted into binary images with various thresholds, based on image histogram properties (mean and standard deviation of grey scale levels). The ratio between black pixels (pores) and the total number of pixels made it possible to evaluate the porosity of the electrospun scaffolds. Image processing was performed with the free software Scilab (5.4.1, Scilab Enterprises, Orsay, France).

### 4.4. Mechanical Characterization of the Scaffolds

Conventional tensile tests were carried out on the electrospun scaffolds using a Bose Electroforce 3200 device (Bose, Framingham, MA, USA) at the speed of 0.1 mm/s until plastic deformation was attained. Strips measuring 5 mm × 30 mm were cut out of large scaffold sheets and mounted on metallic clamps with gripping surfaces after the thickness has been measured with caliper (0.1 mm). The curve force versus displacement was recorded. The Young’s Modulus was calculated from the linear elastic part of the stress-strain curves (Linear regression tool, MS Excel software, Microsoft, Redmond, WA, USA). Tests were conducted on dry and wet (humidification with demineralized water) samples. For the aligned fibers (PCLaligned), deformation was classically applied along the fiber alignment (this is also of interest as we expected the cell alignment in the same direction along the fibers). All the measurements were performed at room temperature.

### 4.5. Wettability

The wettability of the electrospun scaffolds was evaluated with water contact angle measurements using a Krüss DSA10 mk2 device and Drop Shape Analysis software (Krüss GmbH, Hamburg, Germany). Briefly, a droplet of demineralized water was placed on the fiber network with a syringe, and image acquisition was performed immediately. The angle between the horizontal surface and the bottom of the droplet was then estimated with the tangent-2 option of the software. Acute and obtuse angles indicate respectively hydrophilic and hydrophobic behaviors.

### 4.6. Cell Cultures on Electrospun Scaffolds

The same culture medium without lineage-specific additives was used for all the experiments. The murine mesenchymal stem cell line C3H10T1/2 [30] (ATCC CCL-26) initially proliferated in culture flasks at 37 °C, 5% CO_2_, in 1 g/L glucose DMEM medium (Sigma-Aldrich, St. Louis, MO, USA) complemented with 10% of Fetal Bovine Serum (Gibco Invitrogen, Waltham, MA USA), 2% of penicillin-streptomycin (Gibco Invitrogen, Waltham, MA, USA) and 2% of 200 mM l-glutamine (Gibco Invitrogen, Waltham, MA, USA). They were harvested at 95% confluence.

Sodalime glass Petri dishes (40-mm diameter, 12-mm height, Duran, Wertheim, Germany) were precoated with Sigmacote (Sigma-Aldrich, St. Louis, MO, USA) to prevent cell adhesion and thus to promote cell growth specifically on the scaffolds and not on the Petri dishes. Tabs made from polydimethylsiloxane (PDMS) were placed on the dish caps to allow gas transfers with the environment.

The Petri dishes were sterilized by autoclaving (121 °C, 1 bar, 20 min). Scaffold samples (17 × 17 mm) placed in the dishes were immersed in 70% ethanol for 30 minutes and rinsed three times with Phosphate Buffered Saline (pH 7.4 PBS, Gibco Invitrogen, Waltham, MA, USA). 300,000 cells were then seeded on to each sample and cultured for 96 h in the culture medium described above without any additional bone or tenogenic differentiation factors. The medium was changed every 2 or 3 days. Cells were also cultured in dishes without coatings or scaffolds as a material-free control group.

### 4.7. Cell—Material Interactions

After 96 h of culture, some samples were immersed in Rembaum solution [66] for 1 h then rinsed three times with demineralized water. They were observed using SEM (Philips XL30 ESEM-FEG, Eindhoven, The Netherlands; quanta FEG 250, FEI, Hillsboro, OR, USA; Hitachi S-3400N, Tokyo, Japan) after gold coating.

Others were stained to observe the alignment of the actin filaments using fluorescence microscopy. Briefly, samples were fixed in paraformaldehyde (Agar Scientific, Stansted, UK), immersed for 45 min in a rhodamine/phalloidin solution (5 units/mL, Invitrogen, Waltham, MA, USA) then rinsed with PBS. The nuclei of the cells were also stained with DAPI (1 g/L, Invitrogen, Waltham, MA, USA). Samples were then observed (Leica Microsystems, Wetzlar, Germany) with excitation and emission wavelengths of 540/565 nm (rhodamine/phalloidin) and 358/461 nm (DAPI).

### 4.8. Cell Viability

Cell viability was estimated with a Live/Dead^®^ kit (Invitrogen, Waltham, MA, USA) according to the manufacturer’s protocol. Briefly, Calcein AM (1 mM) and Ethidium homodimer-1 (EthD-1, 1 mM) fluorescent dyes were used to stain viable and dead cells, respectively. The samples were observed using fluorescence microscopy (Leica microsystem, Wetzlar, Germany), allowing us to qualitatively determine cell viability and the morphology of the living cells.

### 4.9. Gene Expression Analysis

Gene expression was studied using RT-qPCR (reverse transcription quantitative polymerase chain reaction) after 96 h of culture on the scaffolds. Briefly, samples were lysed with 350 µL of RLT Buffer (Qiagen, Germany) and centrifuged to extract the RNA (ribonucleic acid) according to the manufacturer’s protocol. RNA was transcribed to DNA (deoxyribonucleic acid) using a High Capacity cDNA Reverse Transcription kit (Applied Biosystems, Foster City, CA, USA) according to the manufacturer’s protocol. RT-qPCR was performed using SYBR Green PCR Master Mix (Applied Biosystems). Relative mRNA levels were calculated using the 2^−ΔΔCt^ method [67]. The ΔCts were obtained from Ct normalized with the *Rn18S* or *Rplp0* gene levels in each sample. RNA samples originating from 3 to 8 independent experiments were analyzed in duplicate. The primers are listed in Table 3 and reactions were checked before experiments (efficiency > 80%, R2 > 0.99). The final results were normalized with data from samples cultured without scaffolds, i.e., data were plotted as a ratio to a cell-only control group, highlighting the intrinsic effect of the scaffolds on the gene expression.

### 4.10. Statistic Tests

Six independent experiments were performed for each study. Means and standard deviations were calculated. Two-way analysis of variance with Tukey’s test (mechanical and morphological characterization) and an unpaired *t*-test (gene expression) were used to define the significance of the results.

## 5. Conclusions

In this study, mesenchymal stem cells were cultured on various electrospun fibers under identical culture conditions to investigate the ability of these scaffolds to promote osteogenic or tenogenic cell differentiation. Pure PCL-based scaffolds with 600 or 1000 nm fiber diameters promoted the bone differentiation of stem cells, while blend/mixed materials were more prone to favor tendon differentiation. PLA added to PCL (coaxial blend) and fiber diameter (around 2 µm) were shown to be additional parameters able to participate in a switch from bone to tendon differentiation in the same culture conditions. We conclude that the parameters of the commonly-used method of electrospinning can monitor the stem cell differentiation potential in the absence of specific differentiation factors. These findings are of interest for the development of clinically relevant tissue engineering processes for the regeneration of the musculoskeletal system, and should further be confirmed with long-term studies as well as the use of primary MSCs. With the intrinsic effect of the scaffolds fully analyzed, other microenvironmental signals can be applied to the cell culture to enhance further the stem cell differentiation in chemical factor-free conditions. In particular, mechanical solicitations are widely investigated for bone tissue engineering and can be easily applied to electrospun fiber mats in situ.

## Figures and Tables

**Table 1:** Production parameters of the electrospun scaffolds studied.

Scaffold	Abbreviation	Polymer	Concentration	Solvent	Flow	Speed
					Rate	
**Pure polycaprolactone**	**PCL600nm**	PCL	15 wt/wt %	60 wt % DCM 40 wt % DMF	16,6 µL/min	/
**Random PCL**	**PCL1000nm**	PCL	100 mg/mL	TFE	50 µL/min *	250 rpm
**Pure polylactic acid**	**PLA**	PLA	10 wt/wt %	70% DCM 30% acetone	67 µL/min	50 rpm
**Aligned PCL**	**PCLaligned**	PCL	100 mg/mL	TFE	50 µL/min *	1000 rpm
**PCL/PLA coaxial blend (PCL outside)**	**BlendPCLout**	PCL (133 mg/mL) & PLA (66 mg/mL)		TFE	50 µL/min *	250 rpm
**PCL/PLA coaxial blend (PLA outside)**	**BlendPLAout**			TFE	50 µL/min *	

*: 25 µL/min for each solution. DCM: Dichloromethane, DMF: *N*,*N*-dimethylformamide. TFE: 2,2,2-trifluoroethanol.

**Table 2:** Summary of the main results of morphological, mechanical and biological characterization of the scaffolds seeded with C3H10T1/2 stem cells. Spreading was estimated from the SEM and fluorescence acquisitions as moderate (+), high (++) or very high (+++).

Scaffold	Contact Angle (°)	Young’s Modulus (MPa)		Fiber Diameter (nm)	Porosity µ + δ (%)	Cell Response	
		Dry	Wet			Spreading	Differentiation
**PCL600nm**	123	21	20	665	83.8	+++	Bone
**PCL1000nm**	123	30	36	1159	82.8	++	Bone
**PLA**	124.7	24	43	681	81.3	+	Unclear
**PCLaligned**	127	15	63	1032	82.8	++ Alignment	Bone
**BlendPLAout**	128	32	30	2461	82.7	++	Tendon
**BlendPCLou**	130	60	38	1928	83.1	++	Tendon

**Table 3:** Primer sequences used for the reverse transcription quantitative polymerase chain reaction (RT-qPCR) gene expression study on cultured electrospun scaffolds.

*Gene Symbol*	Gene Name	NCBI Reference Sequence	Primer Sequences
***Aqp1***	Aquaporin1	NM_007472.2	Fwd 5′-CAATTCACTTGGCCGCAATGACCT-3′ Rev 5′-TACCAGCTGCAGAGTGCCAATGAT-3′
***Bglap***	Bone gamma carboxyglutamate protein/Osteocalcin	NM_007541.3	Fwd 5′-CAGCGGCCCTGAGTCTGA-3′ Rev 5′-TTATTGCCCTCCTGCTTGGA-3′
***Col1a1***	Collagen 1 alpha 1	NM_007742.4	Fwd 5′-TGGAGAGAGCATGACCGATG-3′ Rev 5′-GAGCCCTCGCTTCCGTACT-3′
***Dlx5***	Distal-less homeobox 5	NM_010056.3	Fwd 5′-CGTCTCAGGAATCGCCAACT-3′ Rev5′-AGTCAGAATCGGTGGCCG-3′
***Rplp0***	Ribosomal protein, large, P0/36B4	NM_007475.5	Fwd 5′-ACCTCCTTCTTCCAGGCTTT-3′ Rev 5′-CTCCCACCTTGTCTCCAGTC-3′
***Runx2***	Runt related transcription factor 2	NM_001271627.1	Fwd 5′-GGTCCCCGGGAACCAA-3′ Rev 5′-GGCGATCAGAGAACAAACTAGGTTT-3′
***Scx***	Scleraxis	NM_198885.3	Fwd 5′-CCTTCTGCCTCAGCAACCAG-3′ Rev 5′-GGTCCAAAGTGGGGCTCTCCGTGACT-3′
***Tnmd***	Tenomodulin	NM_022322.2	Fwd 5′-AACACTTCTGGCCCGAGGTAT-3′ Rev 5′-AAGTGTGCTCCATGTCATAGGTTTT-3′
***Rn18s***	18S ribosomal RNA	NR_003278.3	Fwd 5′-GGCGACGACCCATTCG-3′ Rev 5′-ACCCGTGGTCACCATGGTA-3′
